# Pan-cancer analysis based on epigenetic modification explains the value of HJURP in the tumor microenvironment

**DOI:** 10.1038/s41598-022-25439-0

**Published:** 2022-12-02

**Authors:** Junwu Li, Jun Zheng, Ronggui Zhang, Weili Zhang, Junyong Zhang, Yuanfeng Zhang

**Affiliations:** Department of Urology, The Second Affiliated Hospital of Chongqing Medical University, Chongqing, 400010 China

**Keywords:** Cancer, Computational biology and bioinformatics

## Abstract

To analyze the expression levels, prognostic value and immune infiltration association of Holliday junction protein (HJURP) as well as its feasibility as a pan-cancer biomarker for different cancers. The Protter online tool was utilized to obtain the localization of HJURP, then the methylation of HJURP in tumors were further explored. Thereafter, the mRNA data and clinical characteristics of 33 tumor types from TCGA database were obtained to investigate the expression and prognostic relationship of HJURP in different tumor types. Finally, the composition pattern and immune infiltration of HJURP in different tumors were detected in Tumor Immune Estimation Resource. HJURP was abnormally expressed in most of the cancer types and subtypes in TCGA database. Also, it was associated with poor prognosis of different cohorts. At the same time, the results also showed that HJURP was related to tumor immune evasion through different mechanisms, including T cell rejection and methylation in different cancer types. Besides, the methylation of HJURP was inversely proportional to mRNA expression levels, which mediated the dysfunctional phenotypes of T cells and poor prognosis of different cancer types. Alternatively, our results indicated that HJURP expression was associated with immune cell infiltration in a variety of cancers. HJURP may serve as an oncogenic molecule, and its expression and immune infiltration characteristics can be used as a biomarker for cancer detection, prognosis, treatment design and follow-up.

## Introduction

Early tumorigenesis may be associated with the abnormal function of centromere and the uncertainty in chromosome separation during cell proliferation^1^. In this process, HJURP is responsible for the loading and assembling of histone H3 variant-centromere protein A (CENPA) at the centromere in a cellular regulatory manner^2^. Usually, they regulate and affect tumor occurrence and development in the cell cycle and cell cloning in different ways^3^. Previous studies have shown that the methylation of HJURP can promote tumor development by reducing cell cycle arrest at G0/G1 phase^4^. Meanwhile, overexpression of HJURP is also found to regulate tumor cell growth in vivo, while tumor cell invasion in vitro predicts the poor prognosis of the disease^5,6^. Tumor occurrence and development are a joint construction of genomic modification at genetic and epigenetic levels^7^. Mutations in the genome can be driven by epigenetic patterns in terms of the changes of gene function and malignant proliferation of cells, and genetic patterns including DNA methylation and histone modifications can also be disrupted by genomic changes^8–10^. The tumor microenvironment (TME) is dominated by tumor cells, which can recruit various immune cells, change the antitumor activity of immune cells, and achieve tumor immune escape by down-regulating the reactivity and pattern of immune cells, and by promoting the apoptosis of relevant immune cells^11–13^. Tumor progression is accompanied by different defense and evasion mechanisms, which may alter the composition pattern of immune cells in TME^11,14,15^. Therefore, different types of immune cells and different periods can respond more accurately through the extent and nature of immune infiltration. Previous studies have suggested that HJURP maintains CENPA on centrioles and participates in chromatin segregation, while HJURP is also involved in DNA replication. HJURP can participate in various cell proliferation-related pathways and promote the proliferation of tumor cells, such as HCC. This is consistent with the fact that HJURP requires CENPA to bind to centrioles during telolobe/early G1 phase, which ensures normal chromosome segregation during mitosis^16^. However, there is no sufficient and effective evidence for the pathogenesis of HJURP in a variety of cancers, and it remains unclear about whether HJURP plays a role in TME, cell cycle progression, tumor treatment and prognosis through a common molecular mechanism in the context of immunocytological action.

Fortunately, thanks to the development and maturity of bioinformatics analysis, researchers can now make systematic explanation of the huge biological genomic information and cell-to-cell interaction. Simultaneously, they can provide objective evidence for cancer diagnosis and prognosis by extracting and analyzing the differences between various cancer clinical information in the database^17–19^. Therefore, more and more pan-cancer studies have been conducted by using different computational tools and online network platforms. This work aimed to explore the differential expression, tumor treatment and prognosis of HJURP in different types of tumor cohorts. Also, the relationship between HJURP and tumor immune infiltration was explored. Our results showed that HJURP expression abnormally elevated in the pan-cancer cohort, which was significantly associated with poorer clinical prognosis. Meanwhile, we found that HJURP knockdown and HJURP mutations were associated with better tumor prognosis. HJURP may participate in the immunogenicity mechanisms of different tumor types, and its immune and mutation characteristics may be the potential biomarker for tumor stage diagnosis and prognosis.

## Materials and methods

### Material collection and data analysis tools

Altogether 113,093 mRNA data were obtained for 33 TCGA-derived tumor types, including 730 normal samples and 10,363 tumor samples. Tumor Immune Dysfunction and Exclusion (http://tide.dfci.harvard.edu/login/) can determine the failure degree of T cells in immunothermal tumors and the enrichment of three T cell suppressive cells in immunocold tumor species. Tumor Immune Estimation Resource (http://timer.comp-genomics.org/) can determine the relationship between tumor cells and immune cells based on the detection and quantification of RNA-seq expression profile data. TISIDB (http://cis.hku.hk/TISIDB/index.php/) is based on five large databases to pre-calculate the gene correlation between immune cell infiltration in various tumors, including lymphocytes, immunomodulators, and chemokines. We confirm that all experiments were performed in accordance with relevant guidelines and regulations. All data were processed in the R language (version 3.6.3), and analyzed by “ggplot2”, “survival”, “pROCmRNA” package.

### Differential expression analysis of HJURP in normal and tumor tissues

To comprehensively analyze the differences in HJURP expression between tumor and adjacent normal tissues in diverse TCGA cancer types, the TIMER2.0, the UALCAN Interactive web and the Gene Expression Profiling Interactive Analysis (GEPIA) algorithm were utilized. Differential expression was considered statistically significant at p < 0.05, < 0.01, and < 0.001.

### Analysis of prognostic correlation

To analyze the prognostic correlations of differential HJURP expression, gene alteration, and treatment outcome, overall survival (OS) in the cohort were analyzed by using KM curves. In the survival analysis of differential HJURP expression between different cancer cohorts, the median expression was set as the expression threshold to divide patient samples into high and low HJURP expression groups. Finally, hazard ratios (HRs), 95% confidence intervals (CIs) and log-rank test p-values were obtained. All HRs were obtained from the Cox proportional hazards regression model based on comparisons between high and low expression groups.

### Analysis of epigenetic methylation

The TCGA methylation module in UALCAN Interactive Network resource was used to analyze the differences in HJURP methylation levels between tumor and paired normal tissues of different TCGA cancer types. The promoter methylation level was indicated by b-value, which ranged from 0 (unmethylated) to 1 (fully methylated). Different b-value cutoffs were considered to indicate hypomethylation (b:0.3–0.25) and hypermethylation (b:0.7–0.5). In addition, the Query module of Tumor Immune Disfunction and Exclusion (TIDE) algorithm was utilized to evaluate the impact of genetic and epigenetic alterations of HJURP on the dysfunctional T cell phenotypes.

### Analysis of tumor immune and immunosuppressive cell infiltration

The TIMER2 server was employed to analyze the correlation between HJURP expression and the infiltration of 6 immune cell types, including B cells, CD8+ T cells, CD4+ T cells, macrophages, neutrophils, and dendritic cells (DCs). Also, the relationship between HJURP expression and tumor infiltration in 4 immunosuppressive cell types known to promote T cell rejection, namely, myeloid-derived suppressor cells (MDSCs), cancer-associated fibroblasts (CAFs), M2 subtypes of tumor-associated macrophages (M2-TAMs), and regulatory T (Treg) cells, was analyzed. Association analysis was performed using the purity-corrected partial Spearman Rho values and statistical significance (p < 0.05). The GraphPadPrism software was utilized for data visualization. Heatmap was used to observe the immune cell infiltration levels in 33 TCGA cancers. The above content visualization is done using the TIMER 2.0 server (http://timer.cistrome.org/) and the TIDE server (http://tide.dfci.harvard.edu/).

### Ethics approval

Since all information from the online database has been deidentified and no personal identifying information was used in our analysis, informed consent was not required in our study.

## Results

### Expression and prognostic ability of HJURP in a variety of tumors

**Figure 1:**
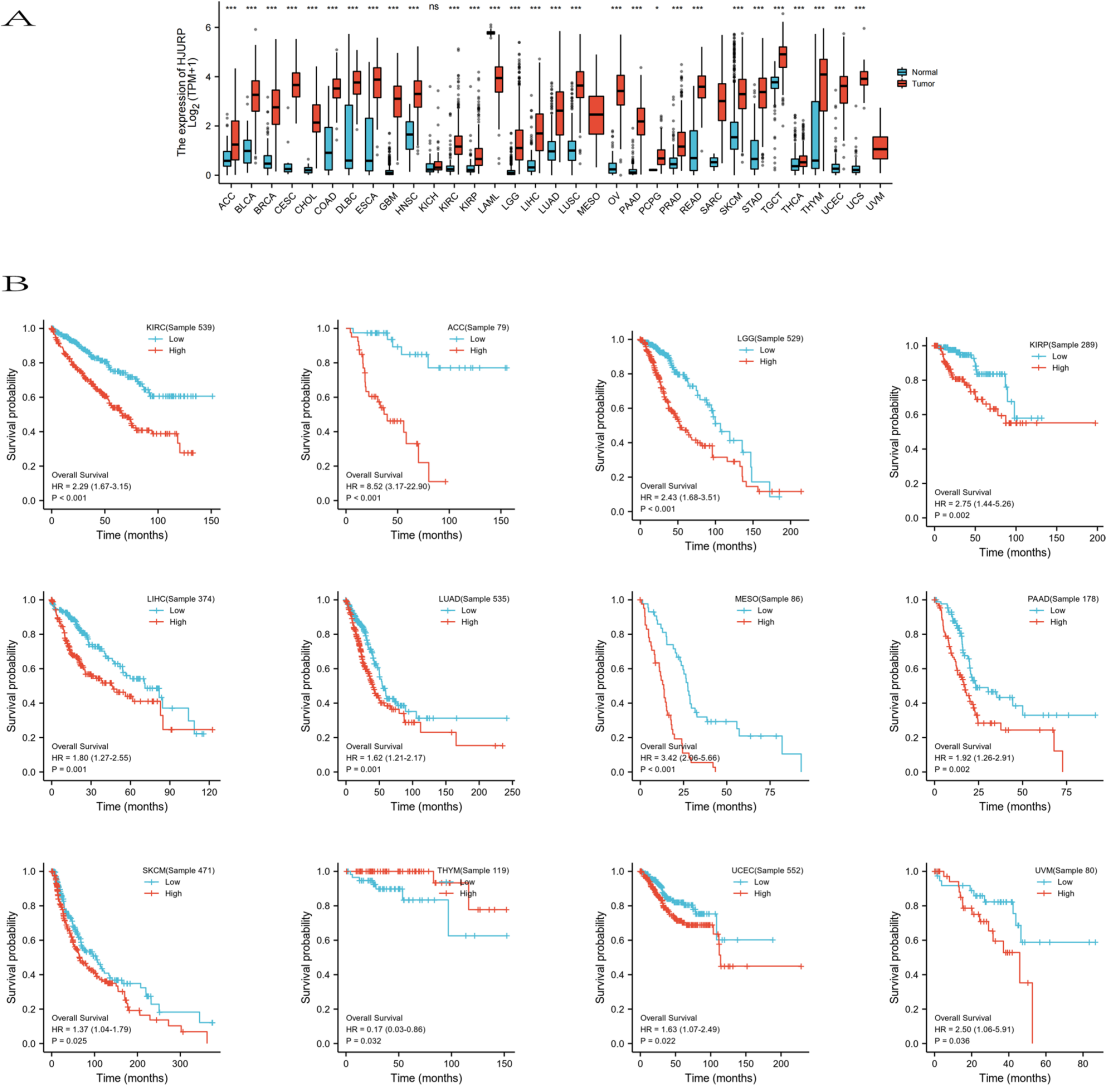
HJURP was significantly overexpressed in most types of tumor tissues (**A**). KM analysis of HJURP expression in KIRC, ACC, LGG, KIRP, LIHC, LUAD, MESO, PAAD, SKCM, THYM, UCEC and UVM (**B**).

The oncogenic effects of HJURP was mined in the entire TCGA pan-cancer database, as a result, compared with normal tissues, HJURP was significantly over-expressed in most types of tumor tissues (p < 0.001) (Fig. 1A). At the same time, correlation analysis was conducted to analyze the relationship between HJURP expression and the prognosis of different tumors by using Kaplan–Meier survival curve analysis. The results suggested a correlation between HJURP expression and tumors such as lung adenocarcinoma, melanoma, diffuse large B lymphoma, renal cell carcinoma, and thymic carcinoma, etc. (Fig. 1B), and patients with high HJURP expression had poorer prognosis. The above results indicated the potential of HJURP as a prognostic and follow-up biomarker for tumors.

### Mutants, localization and functional chaperones of HJURP

**Figure 2:**
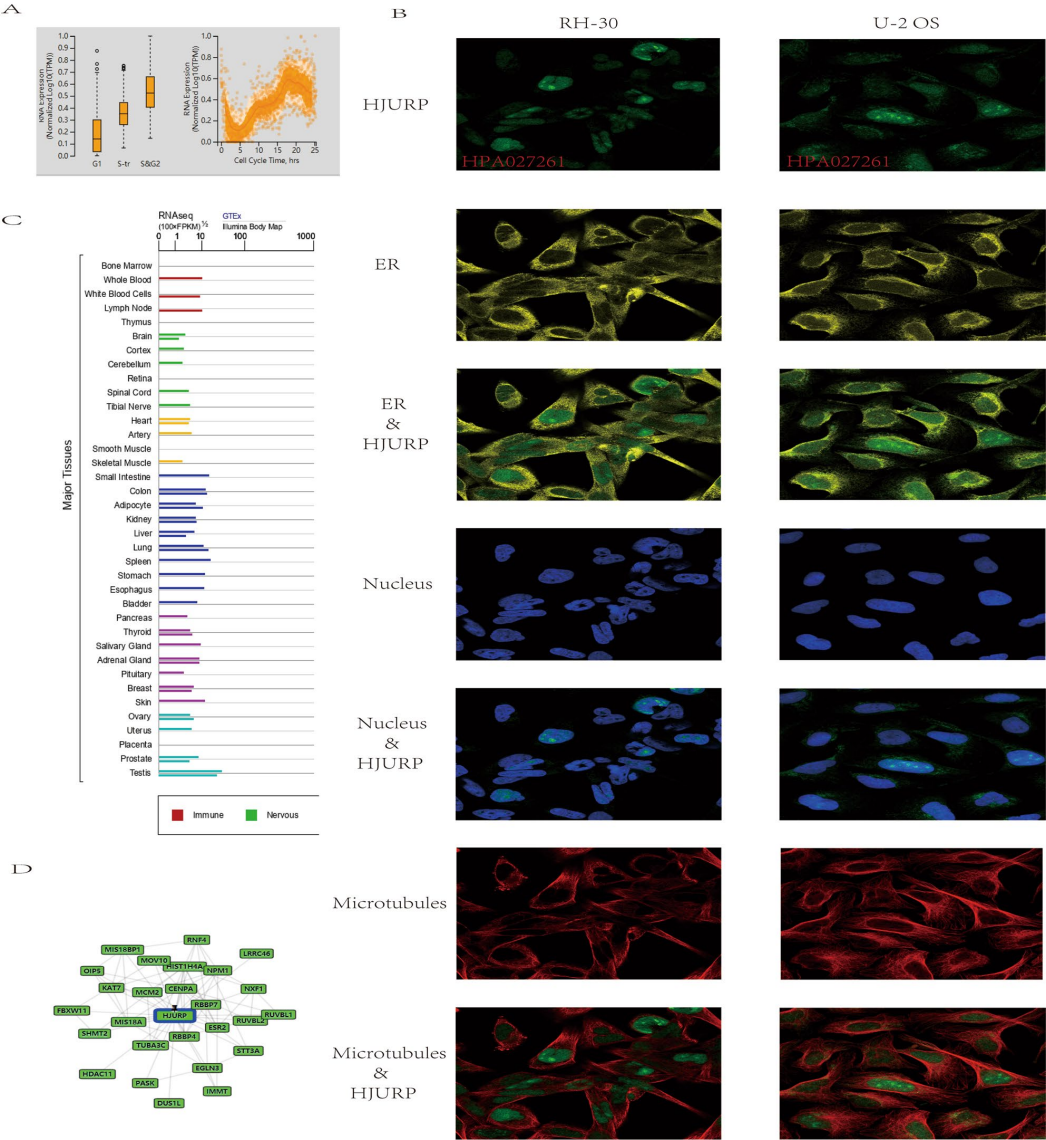
HJURP showed changes in protein expression levels associated with interval progression in G1, S, and G2 phasesa (**A**). HJURP co-localized with nuclear, ER and microtubules markers in RH-30 and U-2OS cells (**B**). mRNA of HJURP is expressed in various normal human tissues (**C**). Gene and disease interaction network analysis of HJURP (**D**).

As shown by our analysis of single-cell RNA sequencing data from fluorescence-based cell cycle index (FUCCI) U-2OS cells, the increased mRNA expression of HJURP was associated with cell cycle progression. HJURP showed changes in its protein expression levels temporally associated with cell cycle progression in G1, S, and G2 phases (Fig. 2A). In addition, HJURP mRNA expression was detected in various normal human tissues, including immune, nervous, endocrine, muscle and reproductive tissues (Fig. 2B). To characterize the intracellular localization of HJURP, the distribution of HJURP and its microtubules in RH-30 and U-2OS cells was analyzed using indirect immunofluorescence. We found that HJURP was co-localized with nuclear, ER and microtubule markers in RH-30 and U-2OS cells, indicating the subcellular localization of HJURP (Fig. 2C). In addition, gene and disease interaction network analysis revealed that HJURP had multiple gene functional partners (Fig. 2D). GO enrichment analysis of HJURP and these gene functional partners revealed that they have immune-related functions. Clustering dendrogram of the top-ranked GO biological processes (GO-BP) showing major pathways such as cellular detection biotic interferon-gamma, neuron death oxidative stress, immune assembly phagocytosis bioprocesses, gland migration morphogenesis duct and interleukin-1 cysteine-type production endopeptidase^16^.

### Epigenetic modifications of HJURP

**Figure 3:**
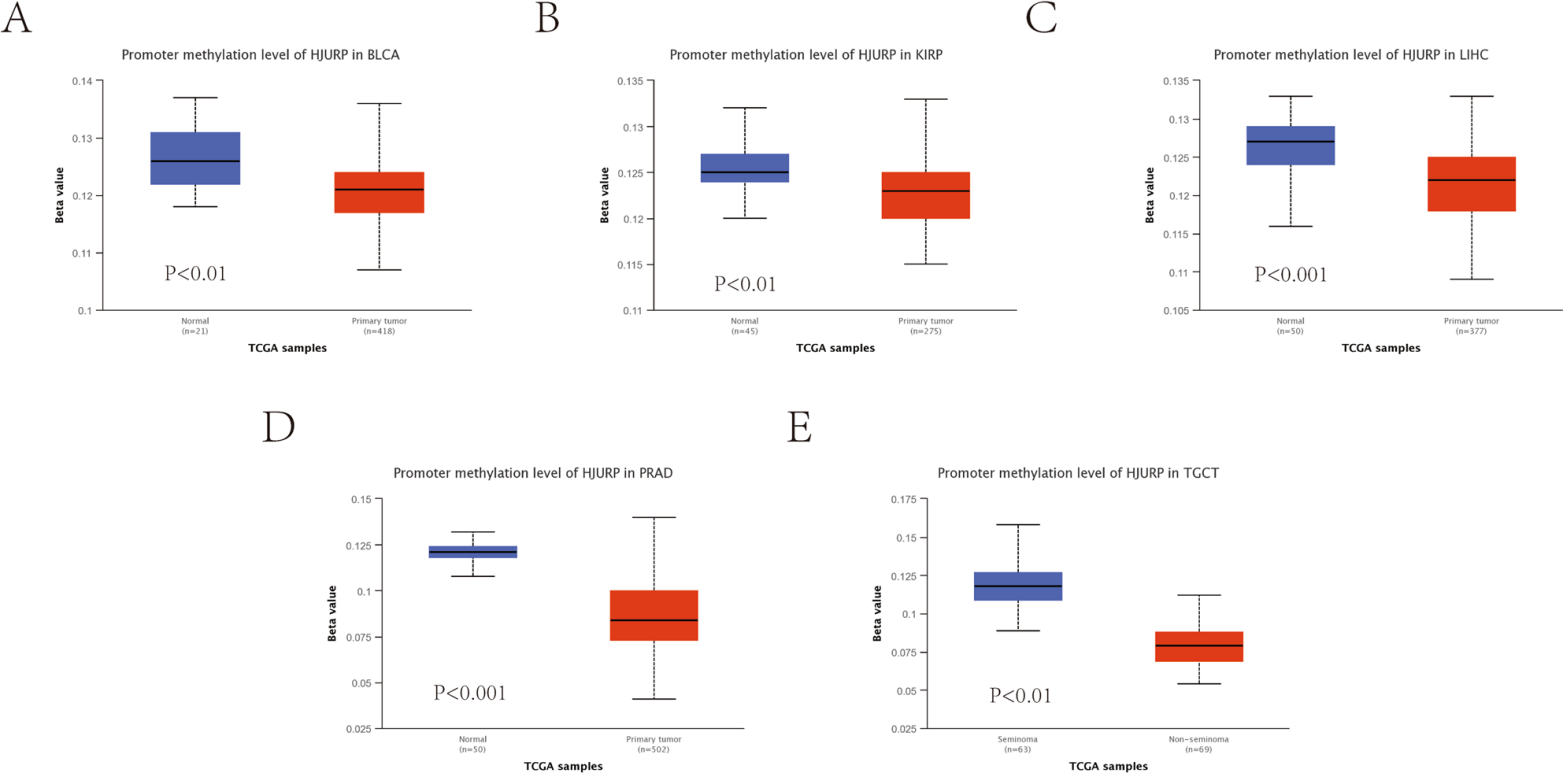
The promoter methylation status chart of HJURP in BLCA, KIRP, LIHC, PRAD and TGCT (**A–E**).

**Figure 4:**
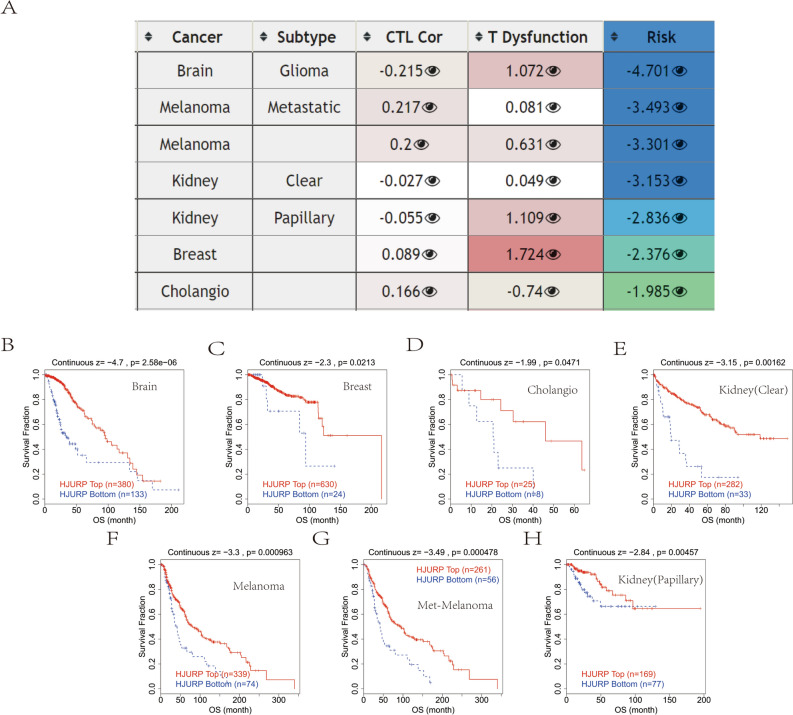
The changes caused by the methylation status of HJURP in different cancers (**A**). HJURP was positively associated with dysfunctional T cells and negatively with survival time in different tumors (**B–H**).

Previous experimental reports have indicated that the hypomethylation of HJURP is highly expressed in tumors, which promotes the progression and migration of tumor cells^4^. As revealed by the promoter methylation status chart, HJURP was hypomethylated in bladder urothelial carcinoma, renal papillary cell carcinoma, hepatocellular carcinoma, prostate cancer and testicular germ cell carcinoma, etc. (Fig. 3A–E). The TIDE algorithm can effectively predict the efficacy of patients on immune checkpoint inhibitors and reflect how tumors suppress the function and infiltration of cytotoxic T lymphocytes (CTL) to achieve immune escape. Numerous studies have found that the level of CTL is associated with better prognosis of patients^20^. If the tumor has a high level of CTL, then the tumor may achieve immune escape by inhibiting the activity of CTL. Conversely, if the tumor has lower CTL levels, it is more likely to achieve immune escape by preventing CTL infiltration. In conclusion, the interactive effect of HJURP expression levels and CTL levels may have an impact on survival. Therefore, the changes caused by the methylation status of HJURP in different cancers were assessed (Fig. 4A). Interestingly, we found that HJURP was positively associated with dysfunctional T cells, but hypomethylation of HJURP was correlated with the shorter OS in different tumors including glioma, melanoma, renal clear cell carcinoma, renal papillary carcinoma, breast cancer and cholangiocarcinoma (Fig. 4B–H). Our results showed that the hypomethylation status of HJURP was associated with poor tumor prognosis.

### Correlation between HJURP and immune cells in infiltration

**Figure 5:**
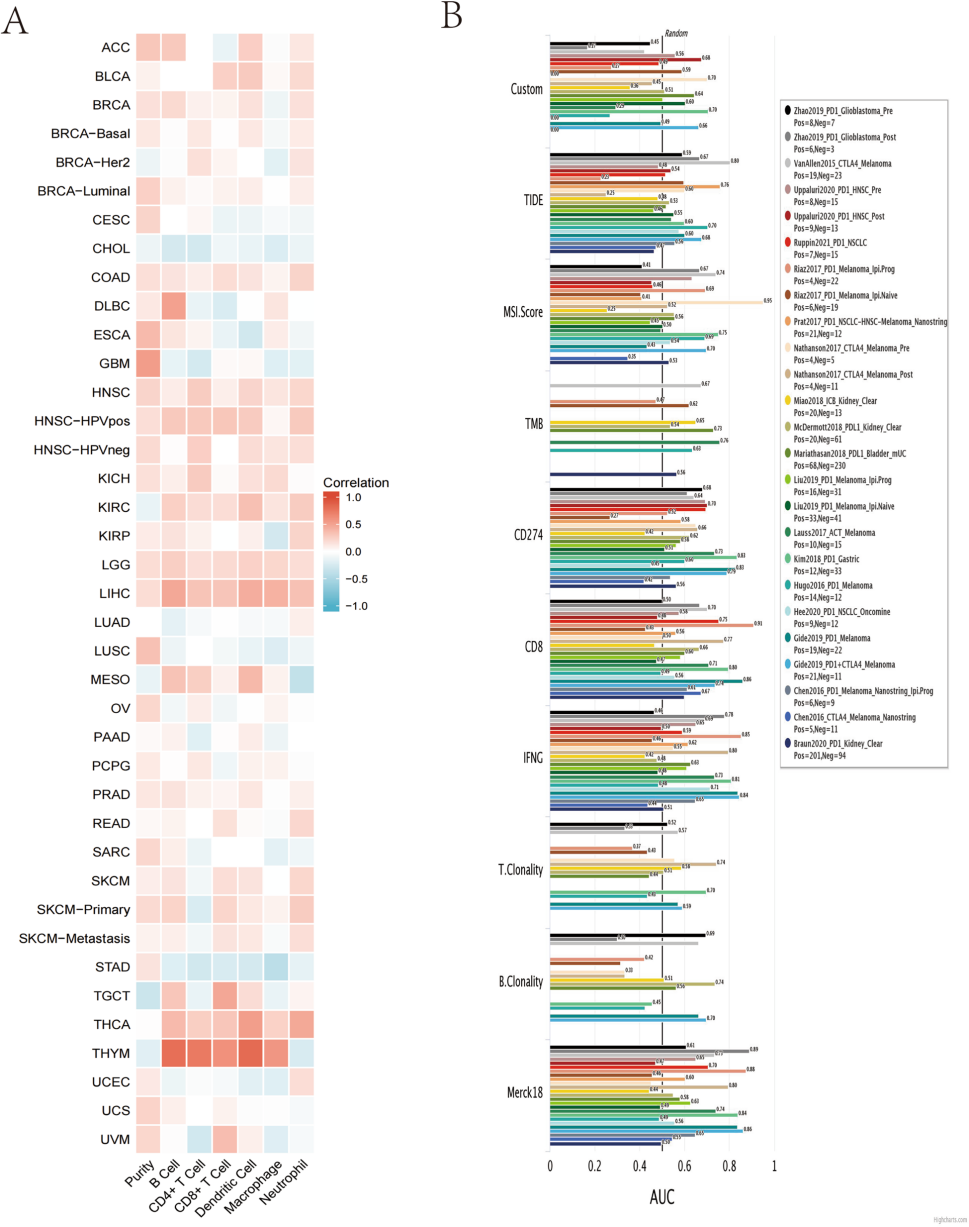
In TCGA cancer types and subtypes of 39 assessing tumor immune infiltration (**A**). The biomarker correlation of HJURP (**B**).

**Figure 6:**
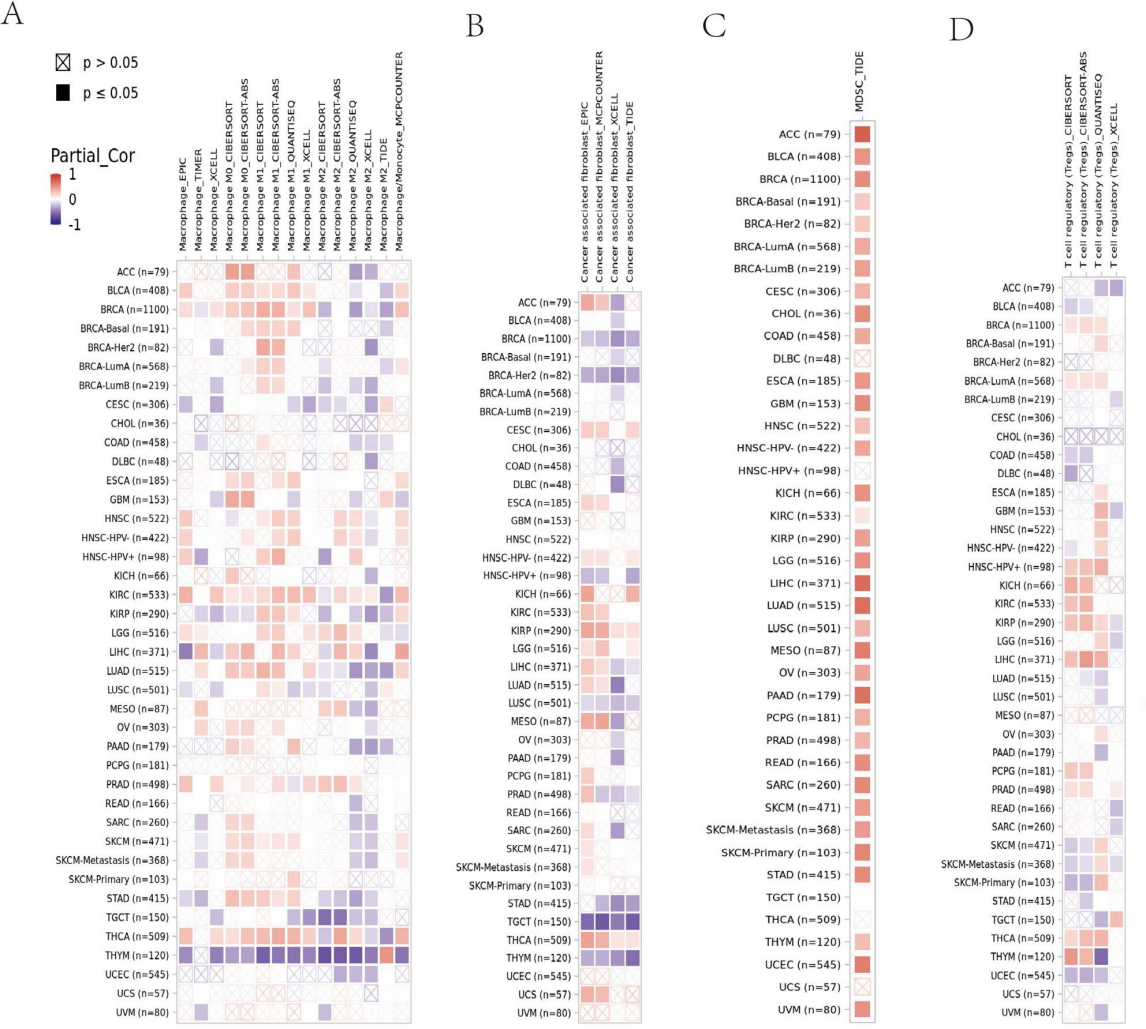
The association of HJURP expression levels with the infiltration of 4 immunosuppressive cells (MDSC, CAF, M2-TAM, and Tregs) (**A–D**).

In 39 TCGA cancer types and subtypes that assessed tumor immune infiltration, BLCA, BRCA, BRCA-Luminal, COAD, HNSC, HNSC-HPVpos, HNSC-HPVneg, KICH, LGG, LIHC and PRAD showed a significant positive correlation between HJURP expression and 7 immune cell types (Purity, B cell, CD4+ T cell, CD8+ T cell, Dendritic cell, Macrophage, Neutrophil) infiltration (Fig. 5A). Moreover, the biomarker correlation of HJURP was assessed by comparing it against the HJURP response results to the ICB subcohort and OS predictive ability to standardized biomarkers. Finally, we found AUC > 0.5 in 18 subjects of the 25 ICB subcohort (Fig. 5B). At the same time, the association of HJURP expression with the infiltration of 4 immunosuppressive cells known to contribute to T cell rejection, namely MDSC, CAF, M2-TAM, and Tregs, was assessed as well. We observed that HJURP expression was associated with ACC, BLCA, BRCA, BRCA-Basai, BRCA-Her2, BRCA-LumA, BRCA-LumB, CESC, CHOL, COAD, ESCA, ESCA, GBM, HNSC, HNSC-HPV-, KICH, KIRC, KIRP, LGG, LIHC, LUAD, LUSC, MESO, OV, PAAD, PCPG, PRAD, READ, SARC, SKCM, SKCM-Metatasis, SKCM-Primary, STAD, THYM, UCEC, and UVMA for MSDC tumor infiltration; CESC, HNSC-HPV−, KIRC, KIRP, LGG, and THCA for CAFs tumor infiltration; BLCA, BRCA, HNSC, HNSC-HPV+, HNSC-HPV−, KIRC, LGG, and THCA for M2-TAM tumor infiltration; and BRCA, HNSC-HPV+, KIRC, PCPG, PRAD, and THCA for Tregs tumor infiltration (Fig. 6A–D).

## Discussion

In this study, based on the protein network map, it was concluded that HJURP was strongly correlated with CENPA and RBBP7, and they both played an important role in the process of cell division in previous studies. The precise diotic inheritance in the mitosis of mammalian nucleated cells is determined by the accurate segregation of chromatin^21^. This process depends on the unique domain of the chromatin. This locus is different from other chromatin in its presence of nucleosome constituted by the histone H3 variant, CENPA. While HJURP participates in CENPA composition in G1 phase of cell cycle and affects the new CENPA recruitment during cell replication^22,23^. Julien et al*.* combined biochemical and quantitative imaging to find that CRL4 is required for CENPA mitophagy loading, and that RBBP7 not only bound and stabilized soluble CENPA during this process, but also served as a specific adapter for the process substrate. The final data concluded that the CRL4-RING E3 ubiquitin ligase complex containing RBBP7 may regulate mitosis by promoting ubiquitin-dependent loading of newly synthesized CENPA during the G1 phase of the cell cycle^24^. Previous research reports have indicated that the overexpression of CENPA is accompanied by the overexpression of HJURP, which leads to ectopic CENPA deposition, thus further leading to mitotic defects, centromeric dysfunction and chromosomal instability^1,25^. Eventually, it leads to the occurrence and progression of cancer. In the study of Heo et al., the cellular senescence regulated by HJURP down-regulation was also found to be mediated via the p53-dependent pathway^26^. In addition to the genetic involvement in cancer development, HJURP also plays an epigenetic role in tumor progression. In previous studies, Lai et al*.* found that HJURP regulated the cyclin-dependent kinase inhibitor 1 (CDKN1A) through the GSK3/JNK signaling pathway in prostate cancer, and affected the growth of tumor cells^27^. Chen et al*.* discovered that HJURP disrupted p21 stability via multiple cellular pathways, including AKT/GSK3, thereby enhancing the HJURP-mediated cell growth capacity^5^. In an in vitro study of breast cancer cell lines by Hu et al*.*, HJURP was reported to adjust the sensitivity of tumor cells to radiotherapy by playing a role in DNA repair^28^. Epigenetic modifications of abnormal HJURP occur at the very early stages of tumor development, and HJURP has functional effects on different types of tumors through different mechanisms of action, which deserve further investigation.

In this study, HJURP methylation was found to be strongly different in most tumors compared to normal tissue, and hypomethylation of HJURP was associated with poorer prognosis in various cancers. Previous studies have shown that HJURP hypomethylation is associated with tumorigenicity, and the results showed that the HJURP gene was significantly hypomethylated in BLCA, KIRP, LIHC, PRAD and TGCT between tumor samples and normal samples. It is reasonable to speculate that HJURP hypomethylation may increase the risk of urological tumor occurrence and development, and the level of HJURP gene and methylation may contribute to the diagnosis and prognosis of urological tumors.

Thanks to the rapid development of the information age, the functional role of HJURP in various cancers has been gradually revealed. For instance, Wang et al*.* identified an association of HJURP with poor OS of NSCLC patients by String database analysis, and HJURP was suggested as a key gene for NSCLC development and prognosis^29^. Fu et al*.* found that HJURP protein expression served as a predictor for lung cancer brain metastasis^30^. Hu et al*.* reported that high HJURP expression was significantly associated with poor OS, tumor number, tumor differentiation, TNM staging, and Barcelona clinical liver cancer staging. Moreover, high HJURP expression was an independent prognostic risk factor for the poor prognosis of liver cancer^28^. In addition, Chen et al*.* discovered that HJURP played an important role in liver cancer metastasis by up-regulating SPHK1, and the high HJURP expression might indicate a lower DFS rate and a higher possibility of microvascular infiltration in HCC patients^31^. By statistical analysis of the Taylor data set, Chen et al*.* found that HJURP up-regulation was significantly related to PSA, high Gleason score, advanced pathological staging, and metastasis^32^. A large number of studies have shown that HJURP exhibits a great potential in pan-cancer research, and the immune-related mechanisms have been clarified in some cancers. Zhang et al. suggested that HJURP is a potential independent prognostic marker for clear cell renal cell carcinoma (ccRCC), which plays an important role in the tumor microenvironment by regulating immune cell infiltration^33^. Luo et al. believed that HJURP is related to tumor-infiltrating immune cells, immune checkpoints and immunosuppression in HCC, and HJURP-related genes involved in immune response may help predict the prognosis of patients^16^. Chen et al. also found significant associations between HJURP and several tumor-infiltrating immune cells, immunomodulators, and immune subtypes in lung adenocarcinoma (LUAD) patients^34^.

The hypomethylation of HJURP mediated T cell dysfunction in 7 tumors and predicted the worse clinical prognosis. As revealed by the results of previous studies, the hypomethylation of HJURP can promote tumor development by reducing the stalling of G0/G1 in the cell cycle. Our results further confirmed the oncogenic role of HJURP methylation in tumors. Tumorigenesis is usually dominated by genetic alterations, while the cumulative effect of epigenetic alterations in HJURP may be important to ultimately drive the invasive development and metastases of tumors. The infiltration of immune cells in tumor can change the function of T cells and further promote tumor escape from the host immune system, leading to tumor progression and metastasis. We explored the immune infiltration patterns of 33 tumors and found that 10 cancer types, including BLCA, BRCA-Luminal, COAD, HNSC, HNSC-HPVpos, HNSC-HPVneg, KICH, LGG, LIHC and PRAD, showed a correlation with immune infiltration of immunosuppressive cells to varying degrees. The infiltration of M2 TAM and MDSC can serve as an objective response to tumor T cell rejection. Surprisingly, we found that the expression level of HJURP was associated with the infiltration of immunosuppressive cells in almost all tumors. These results show that HJURP is most likely to jointly promote tumor metastasis and escape in TME through the infiltration of immune cells and the immune exclusion of T cells, with the immune exclusion of T cells occupying a dominant position. At the same time, the oncogenic role of HJURP was mined in the entire TCGA pan-cancer database. It was found that compared with normal tissues, HJURP expression was significantly higher in most types of tumor tissues and high expression of HJURP was related to poorer prognosis. The above studies strongly suggest the potential of HJURP as a prognostic, and follow-up biomarker for tumors.

Certainly, some limitations should be noted in this study. First, only one TCGA database was selected for the study, which might lead to sample bias. To improve the credibility of the results, the sample size should be further expanded. Moreover, our results should be further validated through experiments in vitro and in vivo, so as to reveal the relevant biological functions of HJURP. In summary, this is the first study to explore the role of differential HJURP expression and its methylation in various tumor cohorts. In addition, this study also deeply explores the relationship between HJURP and tumor immune infiltration, and confirms that HJURP expression and immune infiltration characteristics can be used as the biomarkers for cancer detection and follow-up.

## Data Availability

The datasets generated and analysed during the current study are available in the TCGA database. (https://portal.gdc.cancer.gov).
