# Publisher Correction: Pan-cancer analysis based on epigenetic modification explains the value of HJURP in the tumor microenvironment

**DOI:** 10.1038/s41598-023-37881-9

**Published:** 2023-07-05

**Authors:** Junwu Li, Jun Zheng, Ronggui Zhang, Weili Zhang, Junyong Zhang, Yuanfeng Zhang

**Affiliations:** Department of Urology, The Second Affiliated Hospital of Chongqing Medical University, Chongqing, 400010 China

Correction to: *Scientific Reports* https://doi.org/10.1038/s41598-022-25439-0, published online 02 December 2022

In the original version of this Article, Junwu Li and Jun Zheng were omitted as equally contributing authors.

The original Article has been corrected.

